# Structure and Activity Analysis of Inauhzin Analogs as Novel Antitumor Compounds That Induce p53 and Inhibit Cell Growth

**DOI:** 10.1371/journal.pone.0046294

**Published:** 2012-10-24

**Authors:** Qi Zhang, Derong Ding, Shelya X. Zeng, Qi-Zhuang Ye, Hua Lu

**Affiliations:** 1 Department of Biochemistry and Molecular Biology and Tulane Cancer Center, Tulane University School of Medicine, New Orleans, Louisiana, United States of America; 2 Department of Biochemistry and Molecular Biology, Indiana University School of Medicine, Indianapolis, Indiana, United States of America; 3 Simon Cancer Center, Indianapolis, Indiana, United States of America; National Cancer Center Research Institute, Japan

## Abstract

Identifying effective small molecules that specifically target the p53 pathway in cancer has been an exciting, though challenging, approach for the development of anti-cancer therapy. We recently identified Inauhzin (INZ) as a novel p53 activator, selectively and efficiently suppressing tumor growth without displaying genotoxicity and with little toxicity to normal cells. In order to reveal the structural features essential for anti-cancer activity of this small molecule, we have synthesized a panel of INZ analogs and evaluated their ability to induce cellular p53 and to inhibit cell growth in cell-based assays. This study as described here leads to the discovery of INZ analog **37** that displays much better potency than INZ in both of p53 activation and cell growth inhibition in several human cancer cell lines including H460 and HCT116^+/+^ cells. This INZ analog exhibited much less effect on p53-null H1299 cells and HCT116^−/−^ cells, and importantly no toxicity on normal human p53-containing WI-38 cells. Hence, our results not only unveil key chemical features for INZ activity, but also identify the newly synthesized INZ analog **37** as a better small molecule for further development of anti-cancer therapy.

## Introduction

The p53 tumor suppressor protein can prevent the formation of tumors through several mechanisms, including the activation of cell-cycle checkpoints to prevent damaged cells from proliferation (cell-cycle arrest and DNA repair), the promotion of senescence (permanent cell-cycle arrest), and/or the triggering of cell death (apoptosis or autophagy) [1], [2]. It can also impede cell migration, metabolism, or angiogenesis, which are needed for cancer cell progression and metastasis [1]. Mutations of the tumor suppressor gene *TP53* are detected in ∼50% of all types of human cancers [3], while the functions and stability of the p53 protein are often abrogated via posttranslational mechanisms in the rest of human cancers that contain wild type *TP53* [4], [5]. Therefore, the restoration or reactivation of wild-type p53 function can lead to rapid elimination of tumors. As such, compounds that target the p53 pathway have become promising anticancer drug candidates, and several of them have entered clinical trials [4], [6]. For instance, Nutlin-3 and MI-219 can increase p53 level and activity by interfering with the p53-MDM2 binding [7]–[9]. Even though there have been extensive endeavors to find small molecules that target the p53 pathway, none has yet proven to be clinically effective therapeutics due to the inherent undesirable toxicity to normal cells and tissues.

**Figure 1:**
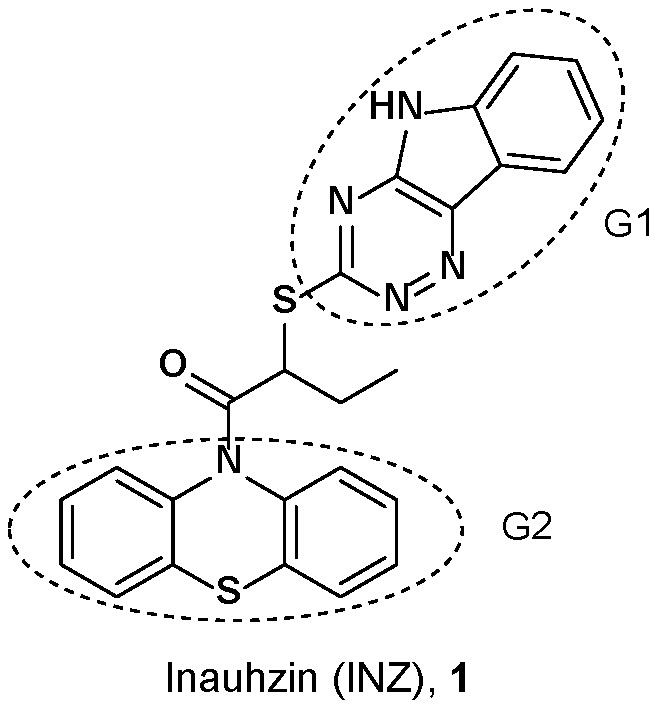
Structure of Inauhzin (INZ).

Through our recent efforts in conducting *in silico* screening and cellular-based assays [10], we discovered Inauhzin (INZ) and its analogs (**Figure 1**) as a novel class of small molecules that effectively activate p53 and promote p53-dependent apoptosis of human cancer cells without causing apparently genotoxic stress. In addition, INZ stabilized p53 by increasing p53 acetylation and preventing MDM2-mediated ubiquitylation of p53 in cells. Remarkably, INZ inhibited cell proliferation, induced senescence and tumor-specific apoptosis, and repressed the growth of xenograft tumors derived from p53-harboring lung cancer H460 and colon cancer HCT116^+/+^ cells without causing apparent toxicity to normal tissues.

INZ is an effective anti-cancer agent either alone or in combination with Nutlin treatment [11] or DNA damage agents Cisplatin and Doxorubicin (unpublished). Single treatment with Nutlin-3 is less efficient in inhibiting the growth or promoting apoptosis of some cancer cells, such as HCT116^+/+^, H460, or A549, in xenograft tumor models even though these cells contain wild type p53. Combination of INZ with Nutlin-3 synergistically promotes apoptosis in HCT116^+/+^ and H460 cell lines in a p53-dependent fashion. This combination also synergistically activates p53 in xenograft tumors derived from these cancer cells and significantly suppresses their growth.

To further characterize the structural features essential for the activity of this group of small molecules to induce p53 and to suppress cell proliferation, we initiated structure-activity relationship (SAR) analyses of INZ analogs. To this purpose, we synthesized a number of new INZ analogs and also evaluated their capability of p53 induction and cell growth inhibition using cell-based assays. Our study not only reveals critical chemical groups for INZ activity, but also leads to the discovery of INZ derivative **37** that displays better potency in p53 induction and cancer cell growth inhibition than does INZ.

## Results and Discussion

### Design and Chemical Synthesis

**Figure 2:**
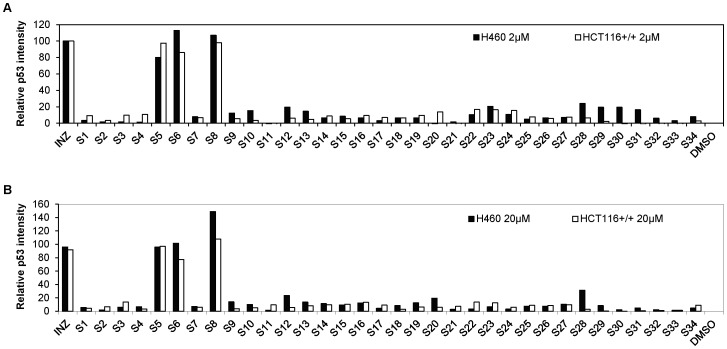
Cellular Activity of INZ Analogs S1–S34. (A–B) Initial Inauhzin analogs were purchased and tested the activity on H460 and HCT116^+/+^ by immunoblotting (IB). Cells were treated with the compounds at 2 µM or 20 µM for 18 hrs and harvested for IB and their p53 induction activity as quantified from IB data as shown in (A–B).

**Figure 3:**
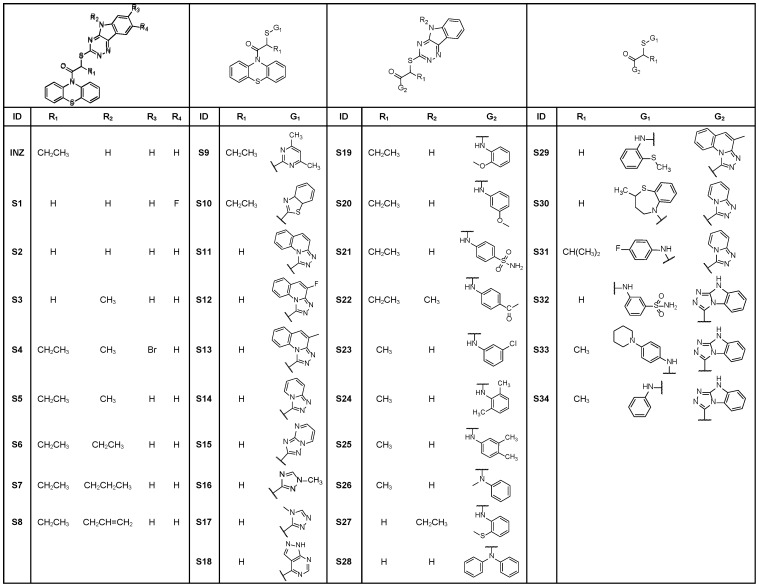
Table of Chemical Structures of Representative Commercial Analogs S1–S34.

**Figure 4:**
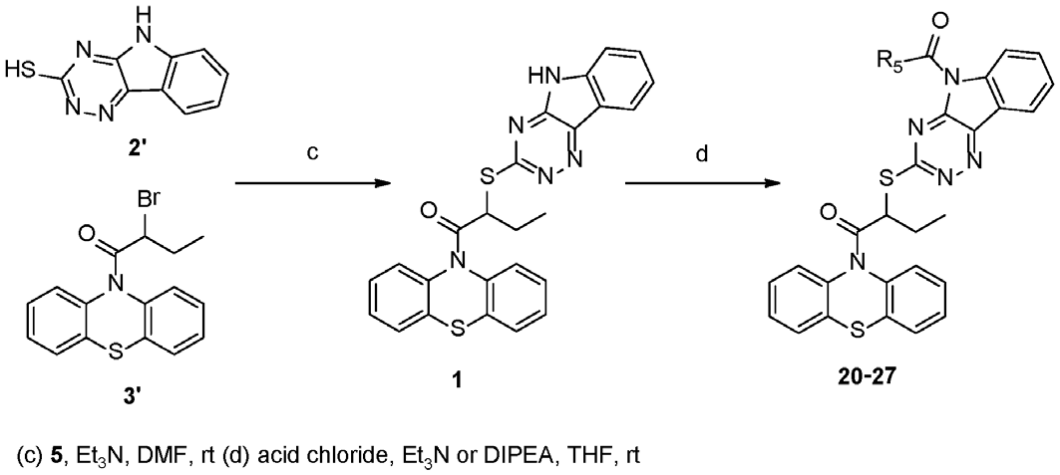
Synthesis of INZ Analogs 20–27.

**Figure 5:**
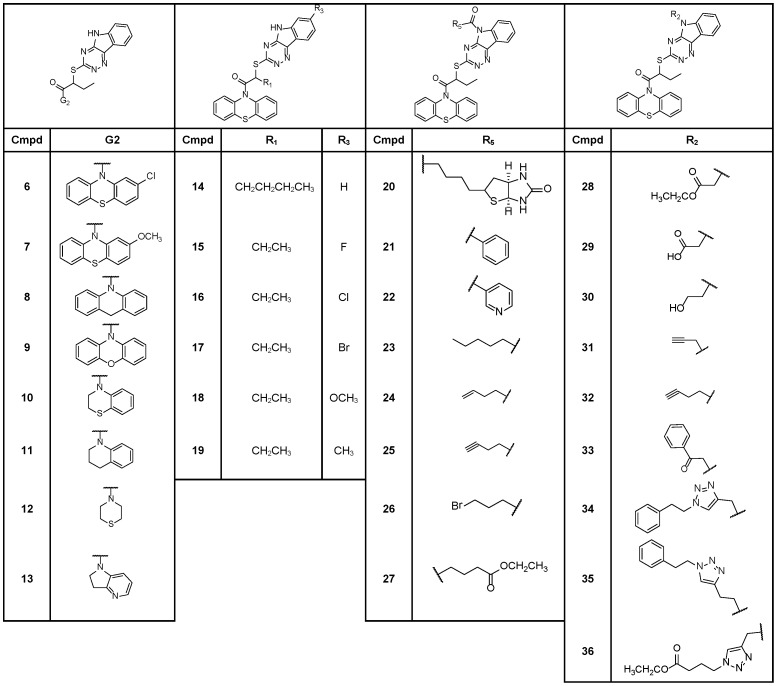
Table of Chemical Structures of INZ Synthetic Analogs 6–36.

Within its structure, INZ (**1**) possesses two distinct chemical components: triazino[5,6-b]indol (G1) and phenothiazine (G2) moiety (**Figure 1**). In our preliminary SAR studies [10], we purchased 46 compounds analogous to INZ with diversities of G1 and G2 and investigated the activity of the compounds in cell-based assays for their ability to induce p53 levels in p53 containing human colon cancer HCT116^+/+^ cells and/or human lung cancer H460 cells using immunoblotting (IB) (**Figure 2** **and** **Figure 3**). The results indicated that a unique structure scaffold might be required for the activity of INZ in cells. Removal of the ethyl group at R_1_ (**S1**–**S3**) or modification at both R_2_ and R_3_ positions on the indol moiety of INZ (**S4**) disabled the compound's ability to activate p53 in cells (**Figure 2**). The R_2_ position can be modified and substituted without loss of activity by replacing it with some alkyl groups, such as methyl, ethyl and allyl, but not propyl (**S5–S8**). Both triazino[5,6-b]indol (G1) and phenothiazine (G2) are essential fuctional groups for p53 induction. The analogs containing ethyl group at the R_1_ position but lacking either functional groups G1 (**S9–S10**), or G2 (**S19–S22**) failed to induce p53. Compounds **S11–S18, S23–S28, and S29–S34** with different aromatic moieties other than triazino[5,6-b]indol at G1 and/or phenothiazine at G2 had very low or no activity. Overall, the results suggest that a specific chemical structure with the intact triazino[5,6-b]indol-3-ylthio)butanoyl]-10H-phenothiazine might be crucial for p53 activation in cells. Indeed, INZ (**1**) displayed more potent p53 activation and anticancer inhibition than either of its component fragments, compound **2′** or **3′** (**Figure 4** **,** and data not shown). It suggests that a synergism is achieved when these two structural units are combined within a single molecule. Therefore, we focused our attention on the structural modifications on the pharmacologically active core: triazino[5,6-b]indol or phenothiazine. Modifications included extension of cabon chain length on R_1_ (**14**) (**Figure 5**), the substitution on the phenothiazine ring (G2) (**6**–**13**) (**Figure 5**) or on the triazino[5,6-b]indol ring (G1) (**15**–**36**) (**Figure 5**).

The syntheses of these new INZ derivatives are outlined in **Figures 4** **,** **6** **,** **7** **, and** **8** **.**

**Figure 6:**
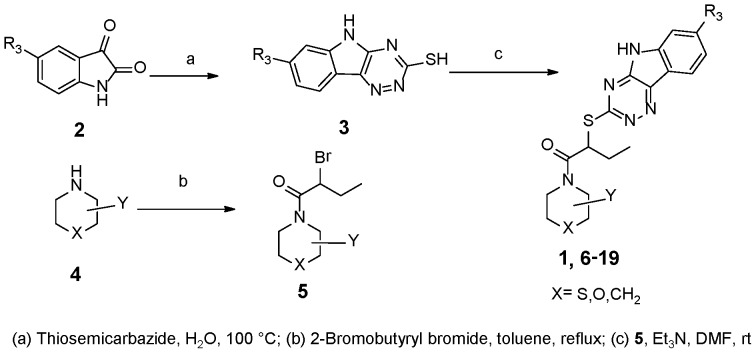
Synthesis of INZ Analogs 6–19.

**Figure 7:**
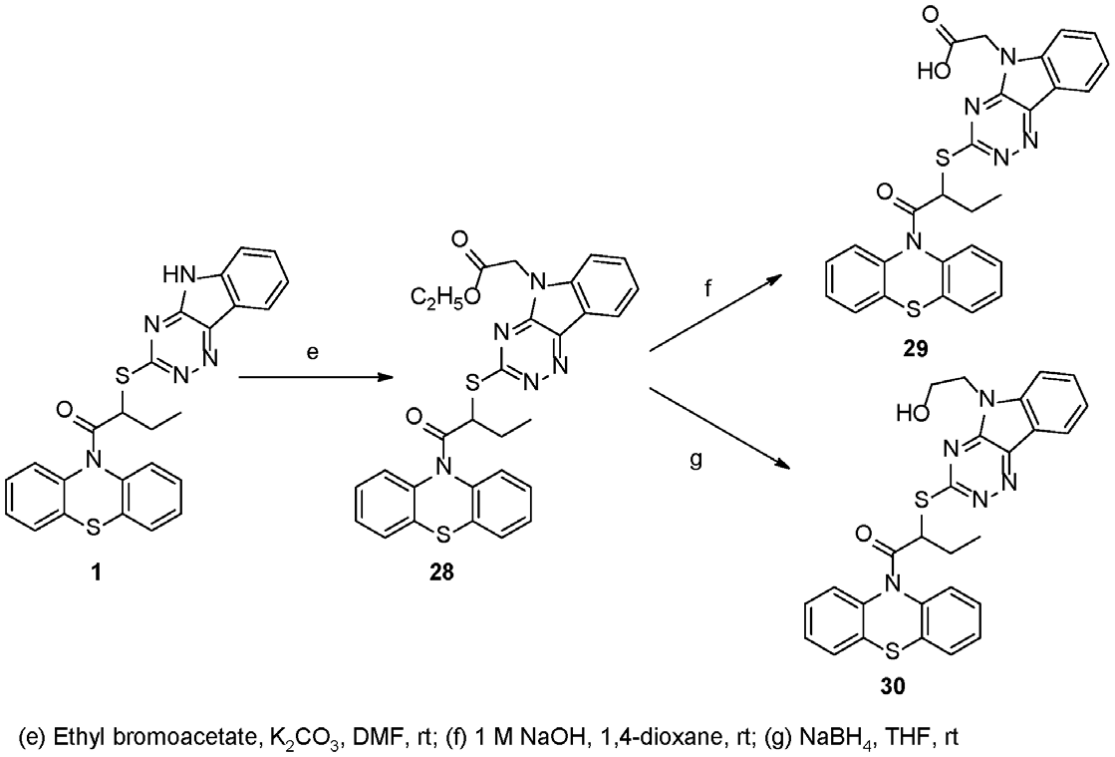
Synthesis of INZ Analogs 28–30.

**Figure 8:**
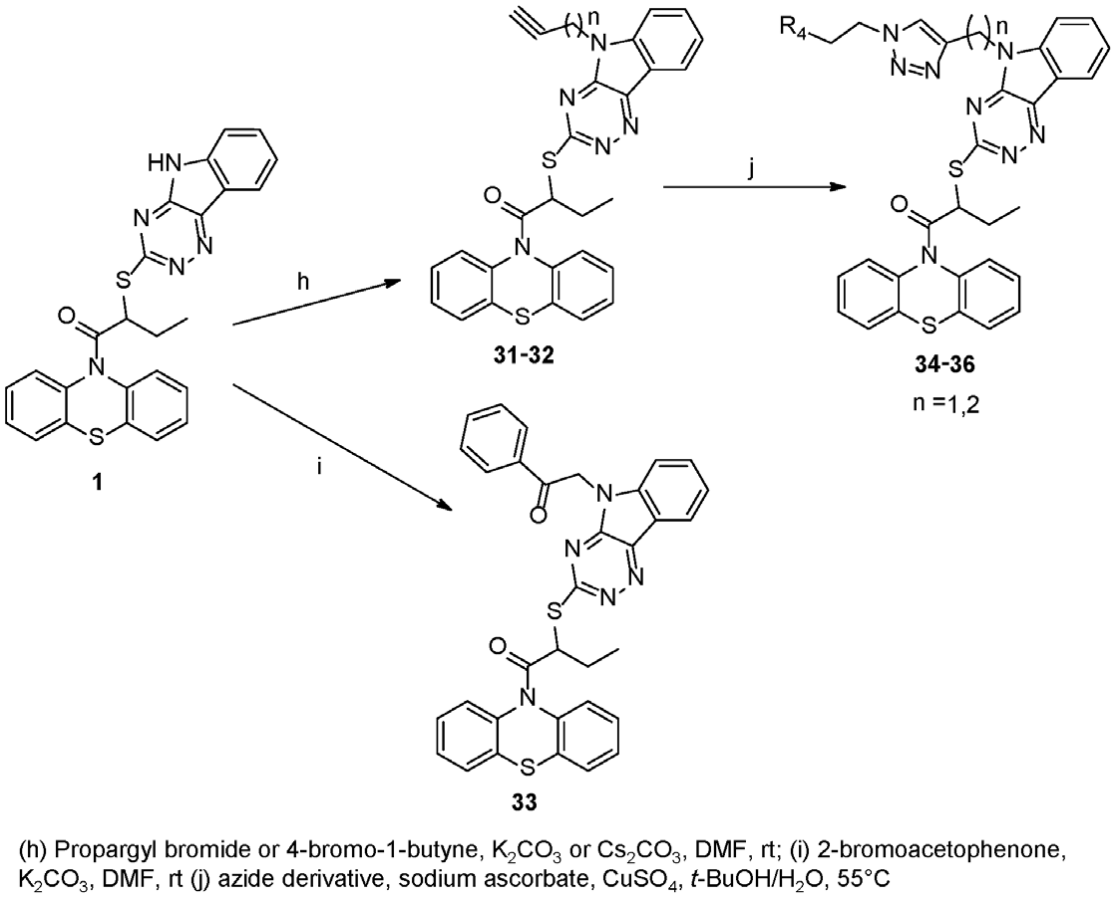
Synthesis of INZ Analogs 31–36.

The synthesis of compounds INZ **(1)** and **6–19** was outlined as **Figure 6**. The 5*H*-[1, 2, 4] triazino[5,6-*b*]indole-3-thiol **3** was prepared from the commercial isatin according to the standard procedure [12]. The bromide **5** was synthesized through refluxed thiophenol with the bromobutyryl bromide in toluene. Then the thiol **3** was reacted with bromide **5** in the presence of Et_3_N and afforded compound **1**, and **6**–**19**. Other bases were tested and some byproducts were produced, which gave rise to low yields.

The amide derivatives **20–27** were prepared in one step from INZ (**1**) in the presence of organic bases as depicted in **Figure 4**.

The amine derivative **28** was synthesized from INZ (**1**) and ethyl bromoacetate in the presence of K_2_CO_3_, which was depicted in **Figure 7**. Other organic bases, such as Et_3_N or DIPEA, were tested and the reaction proceeded very slowly with low yields. Compound **28** was hydrolyzed by 1 M NaOH and afforded the acid **29**. The alcohol **30** was obtained through reduction of **28** by NaBH_4_. LiBH_4_ was tested and several byproducts were produced as revealed by TLC analysis. **Figure 8** shows the “click chemistry” for the synthesis of triazol derivatives. Triazols **34**–**36** were obtained in good yields through the reaction of azide derivative and the propargyl **31** and **32** under the standard conditions [13].

### Biological assessments of INZ analogs

**Figure 9:**
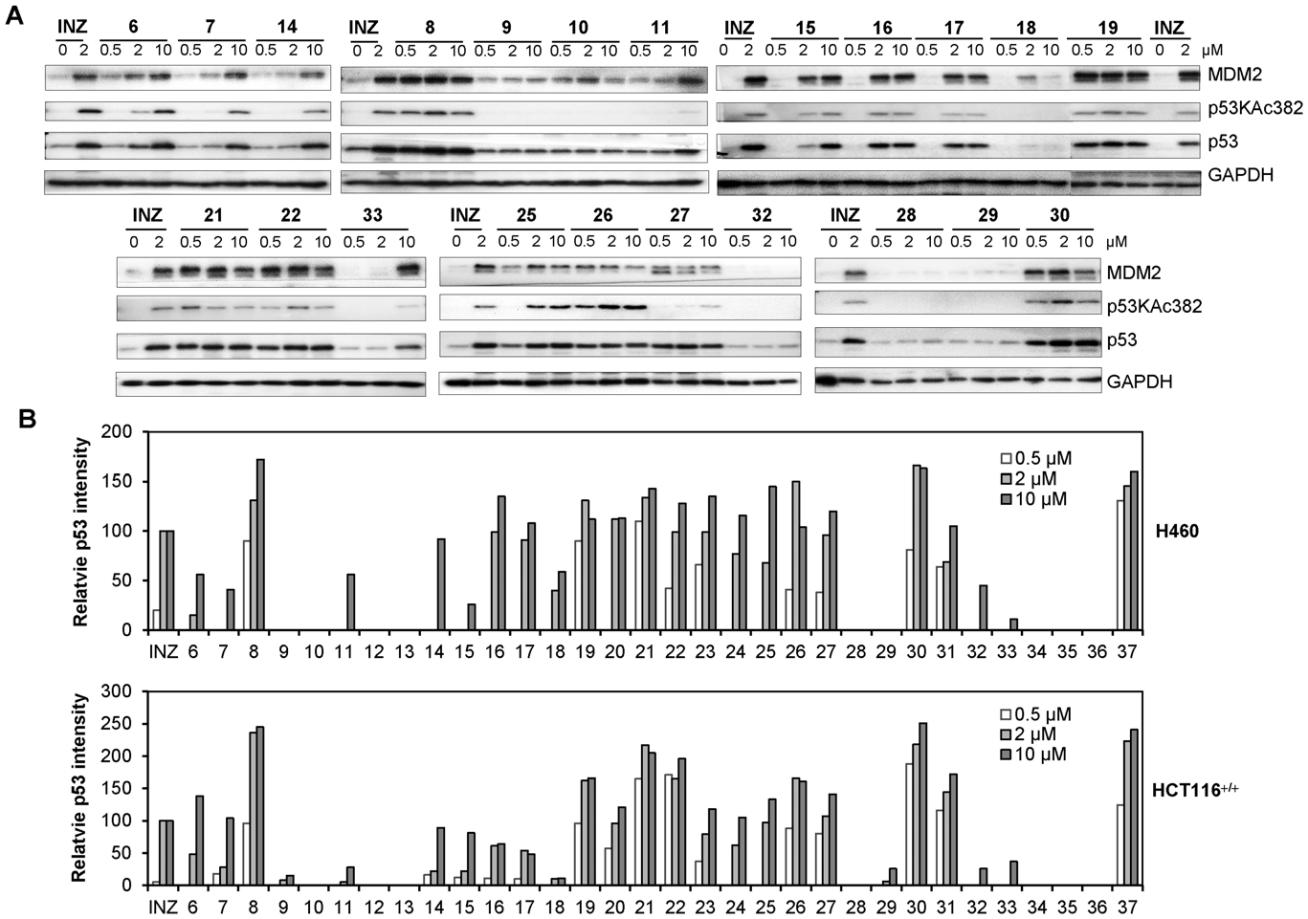
Cellular Activities of INZ Synthetic Analogs 6–37. (A–B) Cellular activity of INZ synthetic analogs **6–37** measured using IB that detects p53 levels and activity in H460 and HCT116^+/+^ cells. Cells were harvested for IB with antibodies as indicated after being treated with each compound for 18 hrs as shown in representative blots from HCT116^+/+^ cells (A) (number denotes each compound; Inauhzin, INZ), and their p53 induction activity as quantified from IB data as shown in (B).

**Figure 10:**
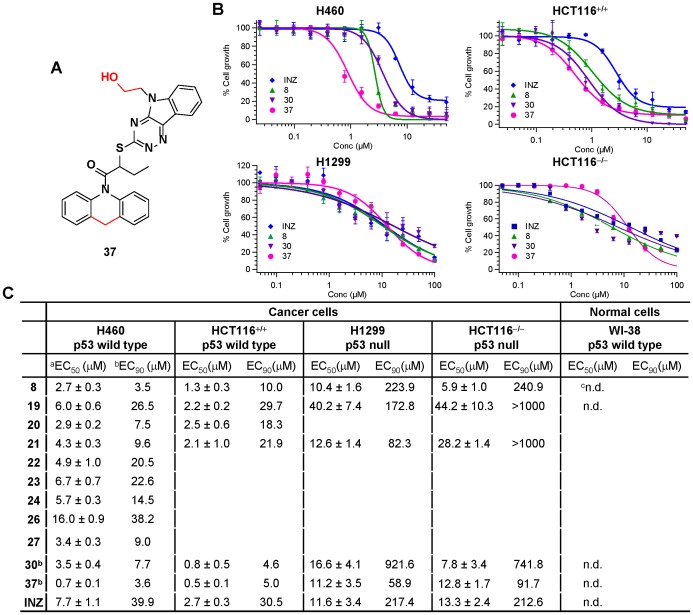
Cell Growth Inhibition by INZ Synthetic Analogs. (A) Structure of INZ synthetic analog **37.** (B) Representative cell growth inhibition curves of INZ synthetic analogs **8**, **30** and **37** in H460, H1299, HCT116^+/+^ and HCT116^−/−^ cell lines. (C) ^a^ EC_50_ of the selected INZ analogs represent the average of triplicates. The EC_50_ values were determined by the two-parameter Hill equation where EC_50_ and the Hill coefficient were allowed to refine while the maximal and minimal values remain fixed. ^b^ EC_90_ values were calculated from the EC_50_ and Hill slope by a web-based calculator: http://www.graphpad.com/quickcalcs/Ecanything1.cfm. ^c^ Not be able to be determined.

The synthetic analogs were then assayed for their potential to induce p53 level and activity in H460 cells and HCT116^+/+^ cells by IB. Compounds were added into cultured H460 and HCT116^+/+^ cells at 0.5, 2, 10 µM for 18 hrs and harvested for IB. The p53 activation was assessed by up-regulating the levels of MDM2, p53 and p53 acetylation. The induction level of p53 by each of the tested INZ analogs was normalized against the loading control of GAPDH and compared to the level of p53 in the cells treated with 2 µM INZ (**Figure 9**). Compounds showing good efficacy in p53 induction were further subjected to a 3-day WST assay to assess their ability to kill cancer cells. INZ was used along with the analogs as a positive control in each assay. The EC_50_ values for their ability to inhibit cell growth were calculated through serial dilution of their concentrations with the highest concentration at 50 µM in H460 and HCT116^+/+^ cells or 100 µM in H1299 and HCT116^−/−^ cells. Four-parameter or two-parameter Hill equation was employed to calculate and plot the dose-response curves as shown with some representative compounds in **Figure 10**. EC_90_ values were calculated from the EC_50_ and Hill slope by a web-based calculator: http://www.graphpad.com/quickcalcs/Ecanything1.cfm.

### Anti-proliferative Effect of Synthetic INZ analogs

In synthetic INZ analogs containing triazino[5,6-b]indol (G1), subtle and major modifications to phenothiazine ring (G2) generally led to less potent molecules. Though subtle changes on the branches of the phenothiazine ring were tolerated (for instance, compounds **6** and **7** with chlorine or methoxy remained active in p53 induction) (**Figure 5** and **Figure 9**), they did not reach 50% p53 induction in H460 cells and HCT116^+/+^ cells at 2 µM. The removal of any ring of G2, as shown for compound **10**–**13** (**Figure 5**), caused loss of activity, and those compounds were essentially inactive (**Figure 9**). The exception to this trend was substitution of the sulfur atom with methylene (**8**). 1-acridin-INZ derivative (**8)** drastically induced p53 at 0.5 µM, whereas compound **9**, whose sulfur was substituted with oxygen, was inactive (**Figure 9**). It should be noted that 1-acridin-INZ (**8**) also exhibited more than 2 fold higher potency than did INZ in its inhibitory effect on H460 (EC_50_ = 2.7 µM) (**Figure 10**) and HCT116^+/+^ cells (EC_50_ = 1.3 µM) (**Figure 10**). The EC_90_ values of this analog were in the range of 3.5–10 µM, which were 3–10 fold lower than those for INZ.

Compound **14** (**Figure 5**) with the longer chain containing butyl at R_1_ position exhibited lower activity for p53 induction, which further indicated that the appropriate length of alkyl chain at R_1_ position is crucial for the activity of INZ, as INZ activity in p53 activation was reduced or lost when the chain was either longer than 2 carbons (**14**, **Figure 5**) or removed (**S1**–**S3**, **Figure 3**). Compounds **15**–**19** (**Figure 5**) were synthesized to determine the effect of different substituents, such as electron-withdrawing group (halogen atoms) and electron donating group (methyl or methoxy), at R_3_ position of indole ring (G1) on p53 induction. Compounds **16** and **17**, which have a chlorine and bromine atom, respectively, exhibited similar activity to that of INZ in HCT116^+/+^ cells with a dose-dependent induction of p53 acetylation at lysine 382, p53 protein level and the up-regulation of MDM2 level (**Figure 9**). Compound **18** with a methoxy group displayed a marked decrease in p53 activation. In contrast, the methyl derivative **19** exhibited a significant effect on p53 induction compared to INZ at 0.5 µM. It also inhibited the proliferation of H460 and HCT116^+/+^ with EC_90_ values of ∼20–30 µM, which were 1.5 fold lower than that for INZ (**Figure 10C**). These results indicate that the order of influence of these substituents on the antiproliferative activity of INZ is as follows: CH_3_>Cl>Br>F>OCH_3_.

The results from our preliminary biological screening of INZ analogs (**S5–S8,** **Figure 3**) suggested that R_2_ position could be modified. We conjugated biotin directly to INZ through the formation of the amide bond at the active hydrogen of R_2_ and gained compound **20** (**Figure 5**). This biotin-conjugated INZ was initially designed for target identification studies. To our delight, the biotinylated INZ (**20**) was as effective as INZ in the induction of p53 acetylation and level in both H460 and HCT116^+/+^ cells [10] (**Figure 9B**). Another biotin-conjugated compound derived from the inactive compound **15** was used as a negative control in the target identification screening (data not shown). In addition to compound **20**, some other amide compounds (**21–27**) (**Figure 5**) were made through the same procedure. All these compounds with various ketone substitutions on R_2_ exhibited good activities in p53 induction and cell growth inhibition in comparison with INZ (**Figure 9** and **10**). Derivatives **20**, **21** and **27** showed similar EC_90_ values of 7.5, 9.6 and 9.0 µM, respectively whereas INZ is about 39.9 µM. Removal (**25→32,** **Figure 5**) or separation (**21→33,** **Figure 5**) of the carbonyl group from the indol resulted in a significant decrease in activity (**Figure 9**). Replacing the ketone with an ester (**28**) or carboxylic acid (**29**) resulted in essentially inactive analogs, in striking contrast to its alcohol derivative **30**, which was comparable to compound **8** in p53 activation and cell growth inhibition (**Figure 5**, **9** **,** and **10**). The EC_90_ values of compound **30** as tested in H460 and HCT116^+/+^ cells, respectively, were ∼7.7 µM and 4.6 µM, which was 5 fold lower than that of INZ (**Figure 10**). Since compounds **8** and **30** displayed more potent activity compared to INZ, we synthesized the analog **37** that contains both substitution of the sulfur atom with methylene on G2 and alcohol substitution on G1. We found that compound **37** was remarkably 10- and 5-fold more active than was INZ in growth inhibition of H460 and HCT116^+/+^ cells (EC_50_ = 0.7 µM and 0.5 µM), respectively.

INZ displayed much higher toxicity to p53-containing human cancer cells than to p53-null cancer cells. This was evident in the EC_50_ and EC_90_ values, which were 1.5 and 5–7 fold greater in p53-null cells than in p53-containing cell lines, respectively (**Figure 10C**). We further examined the activity of INZ synthetic analogs by conducting in vitro cytotoxicity assays using p53 null lung cancer H1299 cells and colon cancer HCT116^−/−^ cells. Compounds **8**, **19**, **21**, **30** and **37** were demostrated much less effective in H1299 cells and HCT116^−/−^ cells, in contrast to their inhibitory activity on p53-containing cells (**Figure 10C**), as the EC_50_ values of compounds **8, 30** and **37** on H1299 were 10.4, 16.6 and 11.2 µM, respectivly, which were 3–15 fold higher than those on H460 cells. The EC_90_ values of compound **8, 30 and 37** on H1299 cells and HCT116^−/−^ cells were greater than 50 µM whereas those on H460 and HCT116^+/+^ cells were 3.5 and 10, 7.7 and 4.6, and 3.6 and 5.0 µM, respectivly. More remarkably, these synthetic analogs were much less toxic to normal human fiberbrast cell WI-38 (**Figure 10C**), while they were much more potent than was INZ in killing p53-containing cancer cells. For example, the EC_50_ value of compound **37** for WI-38 was unable to be determined at the highest concentration tested (50 µM) in comparison of its EC_50_ values of 0.7 and 0.5 µM to p53-containing H460 and HCT116^+/+^ cells, respectively. Together, these results indicate that these more potent INZ analogs, such as compounds **8,** **30 and 37**, possess strong p53-dependent cytotoxicity. Among them, compound **37** stands out as the most effective INZ analog from this study.

## Conclusion

**Figure 11:**
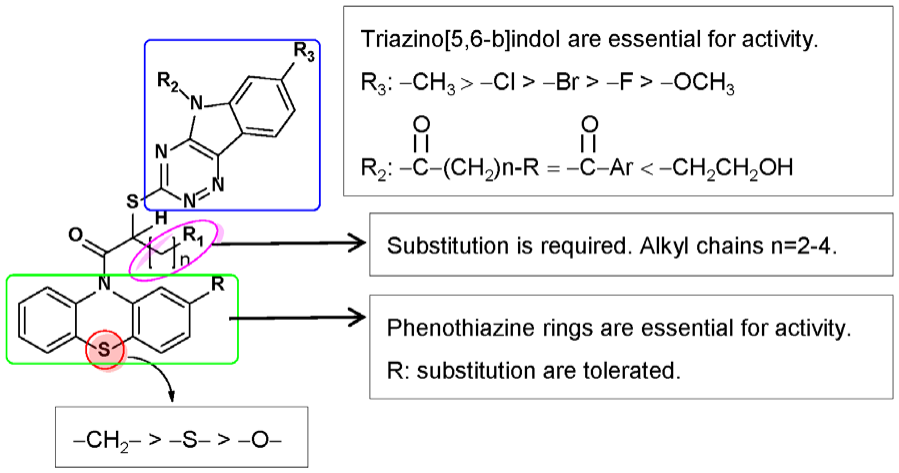
Structure-Activity Relationships of INZ.

Our initial studies on the 46 commercial analogs of INZ yielded information on the important functional groups at each of its two scaffolds indentified as triazino[5,6-b]indol ring (G1) and phenothiazine ring (G2). The functional analyses of the commerical and synthetic analogs of INZ for their ability to activate p53 and to inhibit cell growth further as described above validate that each of the functional groups of INZs is critical for p53 activation and inhibition of cancer cell growth (**Figure 11**). Most modifications to phenothiazine ring G2, such as the branch substitutions (**6**–**7**), or replacement with other rings (**9**–**13, S19–S22**), led to the decreased activity in p53 induction, with the exception of that the substitution of sulfur in the G2 region by methylene (**1→8**) showed greater potentcy than compound **1** in both p53 induction and cancer cell inhibition. Analyses of analogs **S1–S3**, and **14** demonstrate that the ethyl group at R_1_ is required for the activity of these compounds. The butyl group was tolerated. Modification of R_3_ position at the region G1 with methyl, but not halide or methoxy substitutions, increased activity in both of the assays (**15**–**19**). Most modifications on R_2_ at the G1 region resulted in the impressive improvement in terms of p53 activation compared to compound **1 (20–27, 30–31)**. Overall, the best compound from this study was 1-acridin-INZ acohol (**37**). The potency of this analog, compared to INZ, was improved nearly 5- to 10-fold in cancer growth inhibition. Interestingly and importantly, compounds **8**, **30** and **37** were more potent in p53 activation than their parental compound INZ especially with the selective toxicity to p53-containing tumor cells, but not to normal cells.

Based on these SAR and cell-based analyses as described here, 1-acridin-INZ acohol (**37**) represented the most promising candidate for further development and will be selected for further characterization of its biological activity against cancer by using orthotopic lung tumors derived from H460 cells in the near future.

## Materials and Methods

### Compounds S1–S34

INZ analogs **S1–S34** were purchased from Asinex, ChemDiv and ChemBridge. Compounds **S1–S5** were described in a preceding paper, re-validated by LC/MS on an Agilent 1200 LC/MS system (Agilent Technology) at the Chemical Genomics Core Facility of Indiana University School of Medicine. The minimum purity of all compounds is higher than 90%.

### Cell Culture and Immunoblotting Analysis

Human lung carcinoma H460, non-small-cell lung cancer H1299, human colon cancer HCT116 (HCT116^+/+^), and human embryonic fibroblast WI-38 were bought from the American Type Culture Collection (ATCC). Human colon cancer HCT116 p53 null cell lines (HCT116^−/−^) were generously offered by Dr. Bert Vogelstein (Johns Hopkins University) [14]. Those cell lines were cultured in Dulbecco's modified Eagle's medium supplemented with 10% fetal bovine serum, 100 U per mL penicillin, and 100 U per mL streptomycin. Compounds were dissolved in DMSO and diluted directly into the medium to the indicated concentrations; 0.1% DMSO was used as a control. After incubation with the compounds for 18 h, cells were harvested and lysed in 50 mM Tris-HCl pH 8.0, 150 mM NaCl, 5 mM EDTA, 0.5% NP-40 supplemented with 1 mM DTT and 0.2 mM PMSF. An equal amount of protein samples (50 µg) was subjected to SDS-PAGE and transferred to a PVDF membrane (PALL Life Science). The membranes with transferred proteins were blocked with 1×TBST containing 5% non-fat, dried milk for 1 h at room temperature, and then incubated with anti-p53-acetylated (lys382, Cell Signaling), anti-p53 (mouse monoclonal, DO-1, Santa Cruz), anti-MDM2 (4B11) [15], or anti-GAPDH antibodies (Sigma) followed by a secondary antibody labeled with horseradish peroxidase (Pierce). The blots were developed by an enhanced chemiluminescence detection kit (Thermo Scientific), and signals were visualized by Omega 12iC Molcular Image System (UltraLUM).

### Cell Viability Assay

To assess cell growth, the cell counting kit (Dojindo Molecular Technologies Inc., Gaithersburg, Maryland) was used according to manufacturer's instructions. Cell suspensions were seeded at 3,000 cells per well in 96-well culture plates and incubated overnight at 37°C. Compounds were added into the plates and incubated at 37°C for 72 hrs. Cell growth inhibition was determined by adding WST-8 at a final concentration of 10% to each well, and the absorbance of the samples was measured at 450 nm using a Microplate Reader (Molecular Device, SpecrtraMax M5^e^). EC_50_ values were determined by the Hill equation using Igor 4.01 (Lake Oswego, Oregon, USA). EC_90_ values were calculated from the EC_50_ and Hill slope by a web-based calculator: http://www.graphpad.com/quickcalcs/Ecanything1.cfm.

### General Chemistry

All purchased chemicals were reagent-grade or better. Proton and carbon NMR spectra were recorded on a 500 MHz Bruker Avance II spectrometer. Chemical shifts are reported in δ (parts per million, ppm) with the δ 7.26 signal of CDCl_3_ (^1^HNMR), *δ* 2.50 signal of DMSO-*d*_6_ (^1^H NMR), or δ 77.2 signal of CDCl_3_ (^13^C NMR) as internal standards. All column chromatography was performed using Dynamic Adsorbents 230–400 mesh silica gel (SiO_2_) with the indicated solvent system unless otherwise noted. TLC analysis was performed using 254 nm glass-backed plates and visualized using UV light (254 nm). HRMS data were obtained at the Mass Spectrometry Facility at IUPUI Chemistry Department on a Waters/Macromass LCT. All the synthetic compounds were analyzed by LC/MS on an Agilent 1200 LC/MS system (Agilent Technology) at the Chemical Genomics Core Facility of Indiana University School of Medicine and the purity was over 95%.

### General procedure for synthesis of compounds 1, 6–19

#### 2-((5*H*-[1,2,4]triazino[5,6-*b*]indole-3yl)thio)-1-(10*H*-phenothiazin-10-yl)butan-1-one(1, INZ)

2-bromo-1-(10*H*-phenothiazin-10-yl)butan-1-one (3.675 g, 25 mmol) and 5*H*-[1,2,4]triazino[5,6-*b*]indole-3-thiol (2.125 g, 25 mmol) were dissolved in 50 ml anhydrous DMF and cooled to 0°C. 11.1 ml Et_3_N (250 mmol) was dropped to the above mixture. After stirring for1.5 h, TLC indicated that the reaction was completed and stopped. 300 ml ethyl acetate was added to the reaction mixture. The organic phase was washed by saturated NH_4_Cl for five times and separated. It was dried by anhydrous Na_2_SO_4_ and filtered. The organic phase was concentrated to about 15 ml and the pale solid was formed. The amorphous solid was collected and washed by a few ethyl acetate. ^1^H NMR (500 MHz, DMSO-d*_6_*) δ; 12.63 (br, 1H), 8.34 (d, *J* = 7.5, 1H), 7.89–7.96 (m, 1H), 7.56–7.73 (m, 4H), 7.37–7.47 (m, 5H), 7.29–7.22 (m, 1H), 5.27 (t, *J* = 7.0, 1H), 1.86 (br, 1H), 1.74 (br, 1H), 0.85 (br, 3H). ^13^C NMR (125 MHz, DMSO-d*_6_*) δ 170.0, 146.8, 141.7, 140.9, 138.5, 138.3, 131.4, 128.6, 128.2, 128.0, 127.7, 127.4, 123.0, 122.0, 118.0, 113.2, 31.1, 25.9, 11.8. HRMS was calculated for C_25_H_19_N_5_OS_2_ 469.1031 and found 469.1047.

#### 2-((5H-[1,2,4]triazino[5,6-b]indol-3-yl)thio)-1-(2-chloro-10H-phenothiazin-10-yl)butan-1-one (6)

Compound **6** was synthesized similarly to **1**. ^1^H NMR (500 MHz, DMSO-d*_6_*) δ 12.64 (d, *J* = 17.5, 1H), 8.33–8.35 (m, 1H), 7.84–7.91 (m, 1H), 7.60–7.34 (m, 4H), 7.39–7.48 (m, 5H), 5.15–5.26 (m, 1H), 1.82 (br, 1H), 1.73 (br, 1H), 0.85 (br, 3H). HRMS calcd for C_25_H_18_ClN_5_OS_2_ 503.0641; found 503.0643.

#### 2-((5H-[1,2,4]triazino[5,6-b]indol-3-yl)thio)-1-(2-methoxy-10H-phenothiazin-10-yl)butan-1-one (7)

Compound **7** was synthesized similarly to **1**. ^1^H NMR (500 MHz, DMSO-d_6_) δ 12.65(d, *J* = 22.5, 1H), 8.32–8.35 (m, 1H), 7.59–7.73 (m, 1H), 7.36–7.53 (m, 7H), 7.21 (br, 1H), 6.96–7.21 (m, 1H), 5.29 (t, *J* = 7.0, 1H), 3.51–3.71 (m, 3H), 1.95 (br, 1H), 1.77 (br, 1H), 0.86 (br, 3H). HRMS calcd for C_26_H_21_N_5_O_2_S_2_ 499.1137; found 499.1144.

#### 2-((5*H*-[1,2,4]triazino[5,6-*b*]indol-3-yl)thio)-1-(acridin-10(9H)-yl)butan-1-one (8)

Compound **8** was synthesized similarly to **1**. ^1^H NMR (500 MHz, DMSO-d_6_) δ 12.60 (br, 1H), 8.32 (d, *J* = 7.5, 1H), 7.68–7.73 (m, 3H) 7.59 (d, *J* = 8.0, 1H), 7.44–7.47 (m, 1H), 7.17–7.34 (m, 6H), 5.41 (s, 1H), 3.86 (s, 2H), 2.10 (br, 1H), 1.86 (br, 1H), 0.95 (br, 3H). ^13^C NMR (125 MHz, DMSO-d_6_) δ 169.9, 166.1, 146.7, 141.8,140.9, 139.2, 135.2, 131.4, 127.8, 126.7, 126.6, 125.5, 122.9, 122.0, 117.9, 113.2, 47.3, 33.4, 26.2, 11.9. HRMS calcd for C_26_H_21_N_5_OS 451.1467; found 451.1474.

#### 2-((5*H*-[1,2,4]triazino[5,6-*b*]indol-3-yl)thio)-1-(10*H*-phenoxazin-10-yl)butan-1-one (9)

Compound **9** was synthesized similarly to **1**. ^1^H NMR (500 MHz, DMSO-d*_6_*) δ 12.63 (br, 1H), 8.33 (d, *J* = 8.0, 1H), 7.70–7.34 (m, 3H), 7.60 (d, *J* = 8.0, 1H), 7.46 (d, *J* = 7.5, 1H), 7.19–7.25 (m, 6H), 5.47 (t, *J* = 7.0, 1H), 1.99–2.03 (m, 1H), 1.81–1.86 (m, 1H), 0.89 (t, *J* = 7.5, 3H). HRMS calcd for C_27_H_19_N_5_O_2_S 453.1259; found 453.1270.

#### 2-((5*H*-[1,2,4]triazino[5,6-*b*]indol-3-yl)thio)-1-(2*H*-benzo[*b*][1,4]thiazin-4(3*H*)-yl)butan-1-one (10)

Compound **10** was synthesized similarly to **1**. ^1^H NMR (500 MHz, CDCl_3_) δ 10.15 (s, 1H), 8.38 (d, *J* = 7.5, 1H), 7.60–7.66 (m, 3H), 7.43 (t, *J* = 7.5, 1H), 7.23 (br, 1H), 7.12–7.14 (m, 2H), 5.30 (br, 1H), 3.30 (br, 3H), 2.14 (d, *J* = 7.0, 1H), 2.00 (br, 2H), 1.08 (br, 3H). HRMS calcd for C_21_H_19_N_5_OS_2_ 421.1031; found 421.1038.

#### 2-((5*H*-[1,2,4]triazino[5,6-*b*]indol-3-yl)thio)-1-(3,4-dihydroquinolin-1(2*H*)-yl)butan-1-one (11)

Compound **11** was synthesized similarly to **1**. ^1^H NMR (500 MHz, DMSO-d*_6_*) δ 12.60 (br, 1H), 7.69–7.72 (m, 1H), 7.58 (d, *J* = 8.0, 1H), 7.45 (t, *J* = 7.0, 1H), 7.35 (br, 1H), 7.12–7.16 (m, 2H), 7.06 (t, *J* = 7.5, 1H), 5.28 (t, *J* = 6.5, 1H), 3.97 (br, 1H), 3.55 (br, 1H), 2.64–2.76 (m, 2H), 2.04 (br, 2H), 1.86 (br, 2H), 0.89 (br, 3H). HRMS calcd for C_22_H_21_N_5_OS 403.1467; found 403.1479.

#### 2-((5*H*-[1,2,4]triazino[5,6-*b*]indol-3-yl)thio)-1-thiomorpholinobutan-1-one (12)

Compound **12** was synthesized similarly to **1**. ^1^H NMR (500 MHz, CDCl_3_) δ 10.88 (br, 1H), 8.37 (d, *J* = 8.0, 1H), 7.63–7.64 (m, 2H), 7.40–7.43 (m, 1H), 5.35 (t, *J* = 7.0, 1H), 4.12–4.19 (m, 2H), 4.03–4.05 (m, 1H), 3.86–3.89 (m, 1H), 2.92–2.95 (m, 1H), 2.72–2.77 (m, 1H), 2.60–2.64 (m, 2H), 2.19–2.26 (m, 1H), 2.04–2.10 (m, 1H), 1.13 (t, *J* = 7.0,3H). HRMS calcd for C_17_H_19_N_5_OS_2_ 373.1031; found 373.1033.

#### 2-((5*H*-[1,2,4]triazino[5,6-*b*]indol-3-yl)thio)-1-(2,3-dihydro-1H-pyrrolo[3,2-b]pyridine-1-yl)butan-1-one (13)

Compound **13** was synthesized similarly to **1**. ^1^H NMR (500 MHz, DMSO-d*_6_*) δ 12.56 (s, 1H), 8.28 (d, *J* = 8.0, 1H), 8.08 (d, *J* = 4.5, 1H), 7.67–7.71 (m, 2H), 7.56 (d, *J* = 8.5, 1H), 7.42 (t, *J* = 7.5, 1H), 7.00–7.03 (m, 1H), 6.59 (br, 1H), 4.05 (t, *J* = 8.5, 2H), 3.12 (q, *J* = 7.5, 2H), 2.10–2.15 (m, 1H), 1.98–2.04 (m, 1H), 1.07 (t, *J* = 7.5, 3H). HRMS calcd for C_20_H_18_N_6_OS 390.1263; found 390.1274.

#### 2-((5*H*-[1,2,4]triazino[5,6-*b*]indol-3-yl)thio)-1-(10*H*-phenothiazin-10-yl)hexan-1-one (14)

Compound **14** was synthesized similarly to **1**. ^1^H NMR (500 MHz, DMSO-d_6_) δ 12.62 (br, 1H), 8.35 (d, *J* = 7.5, 1H), 7.96 (br, 1H), 7.58–7.74 (m, 4H), 7.37–7.48 (m, 5H), 7.26 (br, 1H), 5.32 (t, *J* = 7.0, 1H), 1.86 (br, 1H), 1.83 (br, 1H), 1.09–1.24 (m, 4H), 0.73 (br, 3H). HRMS calcd for C_27_H_23_N_5_OS_2_ 497.1344; found 497.1348.

#### 2-((7-fluoro-5*H*-[1,2,4]triazino[5,6-*b*]indol-3-yl)thio)-1-(10H-phenothiazin-10-yl)butan-1-one (15)

Compound **15** was synthesized similarly to compound **1**. ^1^H NMR (500 MHz, DMSO-d*_6_*) δ 12.70 (br, 1H), 8.17 (d, *J* = 7.0, 1H), 7.89 (br, 1H), 7.56–7.64 (m, 4H), 7.37–7.43 (m, 4H), 7.22–7.25 (m, 1H), 5.26 (t, *J* = 7.0, 1H), 1.95 (br, 1H), 1.74 (br, 1H), 0.85 (d, *J* = 7.0, 3H). HRMS calcd for C_25_H_18_FN_5_OS_2_ 487.0937; found 487.0962.

#### 2-((7-chloro-5*H*-[1,2,4]triazino[5,6-*b*]indol-3-yl)thio)-1-(10*H*-phenothiazin-10-yl)butan-1-one (16)

Compound **16** was synthesized similarly to **1**. ^1^H NMR (500 MHz, DMSO-d*_6_*) δ 12.79 (br, 1H), 8.38 (s, 1H), 7.88 (br, 1H), 7.73–7.79 (m, 1H), 7.49–7.63 (m, 3H), 7.37–7.43 (m, 4H), 7.23 (br, 1H), 5.26 (t, *J* = 7.0, 1H), 1.86 (br, 1H), 1.74 (br, 1H), 0.86 (br, 3H). HRMS calcd for C_25_H_18_ClN_5_OS_2_ 503.0641; found 503.0641.

#### 2-((7-bromo-5H-[1,2,4]triazino[5,6-b]indol-3-yl)thio)-1-(10H-phenothiazin-10-yl)butan-1-one (17)

Compound **17** was synthesized similarly to **1**. ^1^H NMR (500 MHz, DMSO-d*_6_*) δ 12.84 (br, 1H), 8.50 (s, 1H), 7.85 (d, *J* = 8.0, 2H),7.58 (d, *J* = 8.5, 3H), 7.38–7.41 (m, 4H), 7.23 (br, 1H), 5.26 (s, 1H), 1.86 (br, 1H), 1.73 (br, 1H), 0.85 (br, 3H). HRMS calcd for C_25_H_18_BrN_5_OS_2_ 547.0136; found 547.0136.

#### 2-((7-methoxy-5H-[1,2,4]triazino[5,6-b]indol-3-yl)thio)-1-(10H-phenothiazin-10-yl)butan-1-one (18)

Compound **18** was synthesized similarly to **1**. ^1^H NMR (500 MHz, DMSO-d*_6_*) δ; 12.47 (br, 1H), 7.89 (br, 1H), 7.85 (br, 1H), 7.52–7.64 (m, 4H), 7.37–7.43 (m, 3H), 7.32–7.34 (m, 1H), 7.22 (br, 1H), 5.25 (t, *J* = 7.0, 1H), 1.86 (br, 1H), 1.74 (br, 1H), 0.85 (br, 3H). HRMS calcd for C_26_H_21_N_5_O_2_S_2_ 499.1137; found 499.1136.

#### 2-((7-methyl-5H-[1,2,4]triazino[5,6-b]indol-3-yl)thio)-1-(10H-phenothiazin-10-yl)butan-1-one (19)

Compound **19** was synthesized similarly to **1**. ^1^H NMR (500 MHz, DMSO-d*_6_*) δ 12.50 (br, 1H); 8.13 (s, 1H), 7.87 (br, 1H), 7.49–7.61 (m, 5H), 7.37–7.42 (m, 3H), 7.22 (br, 1H), 5.25 (d, *J* = 6.5, 1H), 2.52 (s, 3H), 1.90 (br, 1H), 1.74 (br, H), 0.86 (br, 3H). HRMS calcd for C_26_H_21_N_5_OS_2_ 483.1188; found 483.1196.

### General procedure for synthesis of compounds 20–27

#### 5-(5-oxo-5-(3-((1-oxo-1-(10*H*-phenothiazin-10-yl)butan-2-yl)thio)-5*H*-[1,2,4]triazino[5,6-*b*]indol-5-yl)pentyl)tetrahydro-1*H*-thieno[2,3-*d*]imidazole-2(5*H*)-one (20)

Biotin (100 mg, 0.410 mmol) was placed in 10 ml reaction flask and cooled to 0°C. 2.7 ml SOCl_2_ was added to the flask and allowed to room temperature. The mixture was stirred for 1 h and excess SOCl_2_ was evaporated. The residue was co-evaporated with 5 ml anhydrous toluene for three times to give the biotin acid chloride. The crude acid chloride was dissolved in 5 ml anhydrous THF. INZ (65 mg, 0.138 mmol) was dissolved in 3 ml anhydrous THF and injected to the above solution through syringe. The mixture was cooled to 0°C and 100 µl Et_3_N (0.717 mmol) was dropped to the mixture. The solution was then allowed to room temperature. TLC was used to monitor the reaction. After 11 h, TLC indicated that the reaction was completed. The reaction mixture was diluted with 30 ml ethyl acetate and washed by saturated NaCl for two times. The organic phase was separated and dried by anhydrous Na_2_SO_4_. The organic phase was filtered, concentrated in vacuum and was purified by column (DCM/CH_3_OH, 55∶1). The product was obtained as viscous oil. ^1^HNMR (500 MHz, CDCl_3_) δ 8.69 (d, *J* = 8.5, 1H), 8.40 (d, *J* = 7.5, 1H), 7.92 (br, 1H), 7.75–7.72 (m, 1H), 7.67 (d, *J* = 7.0, 1H), 7.59–7.56 (m, 1H), 7.53 (d, *J* = 3.0, 1H), 7.40 (br, 1H), 7.35–7.29 (m, 3H), 7.18 (br, 1H), 5.60 (d, *J* = 39.5, 1H), 5.42–5.38 (m, 1H), 5.14 (s, 1H), 4.56–4.53 (m, 1H), 4.41–4.37 (m, 1H), 3.48–3.41 (m, 1H), 3.35–3.26 (m, 1H), 3.25–3.23 (m, 1H), 2.98–2.95 (m, 1H), 2.76 (d, *J* = 12.5, 1H), 1.90–1.83 (m, 4H), 1.79–1.75 (m, 1H), 1.70 (br, 1H), 1.61–1.58 (m, 2H), 0.98–0.90 (m, 3H). ^13^C NMR (125 MHz, CDCl_3_) δ 173.1, 170.0, 167.7,163.7,146.7, 142.4, 139.5, 138.5, 138.3, 132.2, 127.7, 127.5, 127.3, 127.0, 126.8, 125.9, 121.4, 119.6, 117.8, 62.0, 60.4, 55.4, 55.3, 40.6, 39.1, 28.5, 28.4, 26.0, 24.2, 11.7. HRMS calcd for C_35_H_33_N_7_O_3_S_3_ 695.1807; found 695.1817.

#### 2-((5-benzoyl-5*H*-[1,2,4]triazino[5,6-*b*]indol-3-yl)thio)-1-(10*H*-phenothiazin-10-yl)butan-1-one (21)

Compound **21** was synthesized similarly to **20**. ^1^H NMR (500 MHz, DMSO-d*_6_*) δ 8.49 (d, *J* = 7.5, 1H), 8.23 (d, *J* = 8.5, 1H), 7.60–7.78 (m, 6H), 7.40–7.55 (m, 4H), 7.17–7.28 (m, 4H), 7.06 (br, 1H), 4.89 (t, *J* = 6.5, 1H), 1.93 (t, *J* = 6.5, 1H), 1.57–1.62 (m, 1H), 0.75 (t, *J* = 7.0, 3H). HRMS calcd for C_32_H_23_N_5_O_2_S_2_ 573.1293; found 573.1298.

#### 2-((5-nicotinoyl-5*H*-[1,2,4]triazino[5,6-*b*]indol-3-yl)thio)-1-(10*H*-phenothiazin-10-yl)butan-1-one (22)

Compound **22** was synthesized similarly to **20**. ^1^H NMR (500 MHz, CDCl_3_) δ 8.97 (s, 1H), 8.91 (d, *J* = 4.0, 1H), 8.49 (d, *J* = 8.0, 1H), 8.41 (d, *J* = 8.5, 1H), 8.03–8.05 (m, 1H), 7.78–7.84 (m, 2H), 7.66 (t, *J* = 7.5, 1H), 7.58 (d, *J* = 7.0, 1H), 7.48–7.51 (m, 2H), 7.34 (br, 1H), 7.26–7.28 (m, 3H), 7.12 (br, 1H), 5.08 (t, *J* = 6.0, 1H), 1.94–1.96 (m, 1H), 1.67–1.70 (m, 1H), 0.83 (t, *J* = 6.5, 3H).^13^C NMR (125 MHz, CDCl_3_) δ 169.9, 167.7, 166.4,153.8, 150.6,146.7, 141.9, 139.3, 138.4,138.2, 137.3, 132.0, 129.6,128.2, 127.7, 127.3,127.2, 127.1, 126.9, 126.7, 126.2, 123.1, 121.9, 119.9, 116.5, 64.4, 25.8, 11.6. HRMS calcd C_31_H_22_N_6_O_2_S_2_ 574.1246; found 574.1257.

#### 1-(3-((1-oxo-1-(10*H*-phenothiazin-10-yl)butan-2-yl)thio)-5*H*-[1,2,4]triazino[5,6-*b*]indol-3-yl)hexan-1-one (23)

Compound **23** was synthesized similarly to **20**. ^1^H NMR (500 MHz, CDCl_3_) δ 8.70 (d, *J* = 8.5, 1H), 8.42 (d, *J* = 8.0, 1H), 7.89 (br, 1H), 7.68–7.76 (m, 2H), 7.51–7.60 (m, 2H), 7.40 (br, 1H), 7.27–7.35 (m, 3H), 7.18 (br, 1H), 5.37 (t, *J* = 7.0, 1H), 3.26–3.41 (m, 2H), 2.13 (br, 1H), 1.81–1.90 (m, 3H), 1.28–1.46 (m, 4H), 0.98–1.00 (m, 6H). HRMS calcd for C_31_H_29_N_5_O_2_S_2_ 567.1763; found 567.1763.

#### 1-(3-((1-oxo-1-(10*H*-phenothiazin-10-yl)butan-2-yl)thio)-5*H*-[1, 2, 4]triazino[5,6-*b*]indol-5-yl)pent-4-en-1-one (24)

Compound **24** was synthesized similarly to **20**. ^1^H NMR (500 MHz, CDCl_3_) δ 8.71 (d, *J* = 8.5, 1H), 8.43 (d, *J* = 7.5, 1H), 7.89 (br, 1H), 7.73–7.77 (m, 1H), 7.68 (d, *J* = 6.5, 1H), 7.58–7.61 (m, 1H), 7.51–7.53 (m, 1H), 7.41 (br, 1H), 7.34 (t, *J* = 7.0, 1H), 7.26–7.28 (m, 2H), 7.18 (br, 1H), 5.90–5.98 (m, 1H), 5.36 (t, *J* = 7.0, 1H), 5.10–5.20 (m, 2H), 3.48–3.53 (m, 1H), 3.37–3.43 (m, 1H), 2.57–2.62 (m, 2H), 2.12 (br, 1H), 1.89 (br, 1H), 0.98 (br, 3H). ^13^C NMR (125 MHz, CDCl_3_) δ 172.5, 169.9, 167.8, 146.7, 142.4, 139.5, 138.6, 138.3, 136.2, 132.2, 128.3, 127.7, 127.4, 127.3, 126.9, 126.8, 125.9, 121.5, 119.6, 117.8, 116.3, 100.0, 45.8, 38.8, 28.2, 26.1, 11.7. HRMS calcd for C_30_H_25_N_5_O_2_S_2_ 551.1450; found 551.1461.

#### 1-(3-((1-oxo-1-(10*H*-phenothiazin-10-yl)butan-2-yl)thio)-5*H*-[1, 2, 4]triazino[5,6-*b*]indol-5-yl)pent-4-yn-1-one (25)

Compound **25** was synthesized similarly to **20**. ^1^H NMR (500 MHz, CDCl_3_) δ 8.73 (d, *J* = 8.5, 1H), 8.43 (d, *J* = 7.5, 1H), 7.86 (d, *J* = 7.0, 1H), 7.74–7.77 (m, 1H), 7.69 (d, *J* = 7.0, 1H), 7.59–7.62 (m, 1H), 7.54 (d, *J* = 7.5, 1H), 7.34–7.40 (m, 2H), 7.24–7.30 (m, 2H), 7.18 (br, 1H), 5.32 (t, *J* = 7.0, 1H), 3.58–3.62 (m, 1H), 3.45–3.52 (m, 1H), 2.72–2.74 (m, 2H), 2.15 (br, 1H), 1.90 (br, 1H), 1.28 (t, *J* = 7.5, 1H), 0.98–1.01 (m, 3H). HRMS calcd for C_30_H_23_N_5_O_2_S_2_ 549.1293; found 549.1294.

#### 5-bromo-1-(3-((1-oxo-1-(10*H*-phenothiazin-10-yl)butan-2-yl)thio)-5*H*-[1, 2, 4]triazino[5,6-*b*]indol-5-yl)pentan-1-one (26)

Compound **26** was synthesized similarly to **20**. ^1^H NMR (500 MHz, CDCl_3_) δ 8.71 (d, *J* = 8.5, 1H), 8.43 (d, *J* = 7.5, 1H), 7.92 (br, 1H), 7.76 (t, *J* = 8.0, 1H), 7.68 (d, *J* = 7.0, 1H), 7.60 (t, *J* = 7.5, 1H), 7.52–7.54 (m, 1H), 7.41 (br, 1H), 7.30–7.36 (m, 3H), 7.19 (br, 1H), 5.39 (t, *J* = 7.0, 1H), 3.31–3.51 (m, 5H), 2.11 (br, 1H), 1.93–1.98 (m, 2H), 1.85–1.91 (m, 4H), 0.98 (br, 3H). HRMS calcd for C_29_H_24_BrN_5_O_2_S_2_ 617.0555; found 617.0543.

#### Ethyl 5-oxo-5-(3-((1-oxo-1-(10*H*-phenothiazin-10-yl)butan-2-yl)thio)-5*H*-[1,2,4]triazino[5,6-*b*]indol-5-yl)pentanoate (27)

Compound **27** was synthesized similarly to **20**. ^1^H NMR (500 MHz, CD_3_Cl) δ 8.70 (d, *J* = 8.5, 1H), 8.42 (d, *J* = 7.5, 1H), 7.91 (br, 1H), 7.74–7.77 (m, 1H), 7.68 (d, *J* = 7.5, 1H), 7.58–7.61 (m, 1H), 7.52–7.53 (m, 1H), 7.40 (br, 1H), 7.30–7.35 (m, 3H), 7.17 (br, 1H), 5.39 (t, *J* = 6.5, 1H), 3.74 (s, 3H), 3.71–3.72 (m, 2H), 3.42–3.52 (m, 2H), 2.42–2.50 (m, 2H), 1.89–2.02 (m, 2H), 0.98 (br, 3H). ^13^C NMR (125 MHz, CD_3_Cl) δ 173.3, 172.5, 169.9, 167.7, 146.6, 142.4, 139.4, 138.6, 138.3, 132.2, 128.3, 127.7, 127.5, 127.3, 127.1, 170.0, 126.8, 125.9, 121.4, 119.6, 117.7, 68.0, 51.8, 38.4, 32.9, 26.0, 19.5, 11.7. HRMS calcd for C_31_H_27_N_5_O_4_S_2_ 597.1504; found 597.1498.

### General procedure for synthesis of compounds 28–37

#### Ethyl 2-(3-((1-oxo-1-(10*H*-phenothiazin-10-yl)butan-2-yl)thio)-5*H*-[1,2,4]triazino[5,6-*b*]indol-5-yl)acetate (28)

Compound **1** (0.1407 g, 0.3 mmol) was dissolved in 5 ml anhydrous DMF. 50 mg K_2_CO_3_ and ethyl 2-bromoacetate (0.2004 g, 1.2 mmol) were added to the above solution. This reaction was stirred at room temperature. After 6 h, TLC indicated there was no starting material remained and the reaction was stopped. 150 ml ethyl acetate was added to the above mixture. The organic phase was washed by saturated NH_4_Cl for five times and separated. The organic phase was dried by Na_2_SO_4_ and concentrated. The residue was purified by flash column chromatography (hexane/ethyl acetate-2∶1) and viscous oil **28** was obtained. ^1^H NMR (500 MHz, CDCl_3_) δ 8.39 (d, *J* = 7.5, 1H), 7.88 (br, 1H), 7.62–7.63 (m, 2H), 7.42–7.45 (m, 2H), 7.29–7.32 (m, 2H), 7.19–7.26 (m, 2H), 7.09 (br, 2H), 5.37 (t, *J* = 7.0, 1H), 4.97 (s, 2H), 4.19 (q, *J* = 7.0, 2H), 2.01 (br, 1H), 1.82 (br, 1H), 1.20–1.23 (m, 3H), 0.89–0.91 (m, 3H). HRMS calcd for C_29_H_25_N_5_O_3_S_2_ 555.1399; found 555.1405.

#### 2-(3-((1-oxo-1-(10*H*-phenothiazin-10-yl)butan-2-yl)thio)-5*H*-[1,2,4]triazino[5,6-*b*]indol-5-yl)acetic acid (29)

Compound **28** (161 mg, 0.2901 mmol) was dissolved in 15 ml 1,4-dioxane and 0.58 ml 1 M NaOH was added to the solution. The reaction mixture was stirred at room temperature. After 14 h, TLC indicated that there was no starting material remained and the reaction was stopped. The pH of the reaction was adjusted to 4∼5 using concentrated HAc. The mixture was extracted by ethyl acetate for three times and the organic phase was combined. The organic phase was dried by anhydrous Na_2_SO_4_ and concentrated under vacuum. The residue was purified by column (DCM/MeOH-60∶1) and the viscous oil **29** was obtained. ^1^H NMR (500 MHz, CDCl_3_) δ 8.42 (d, *J* = 8.0, 1H), 7.94 (br, 1H), 7.62–7.68 (m, 2H), 7.35–7.49 (m, 4H), 7.24–7.30 (m, 3H), 7.07 (br, 1H), 5.40 (t, *J* = 7.0, 1H), 5.01 (s, 2H), 2.03 (br, 1H), 1.84 (br, 1H), 0.92 (br, 3H). HRMS calcd for C_27_H_21_N_5_O_3_S_2_ 527.1086; found 527.1093.

#### 2-((5(2-hydoxyethyl)-5*H*-[1,2,4]triazino[5,6-*b*]indol-5-yl)thiol)-1-(10*H*-phenothiazin-10-yl)butan-1-one (30)

Compound **28** (95 mg, 0.1712 mmol) was dissolved in 4 ml MeOH/THF (3∶1). The solution was cooled to 0°C. Then NaBH_4_ (39 mg, 1.027 mmol) was added to the above solution. The reaction mixture was allowed to room temperature. After 6 h, TLC indicated there was no starting material remained and the reaction was stopped. Acetic acid was used to quench the reaction. The mixture was purified by column (hexane/ethyl acetate (5∶1-3∶1-1∶1) and the viscous oil was obtained. ^1^H NMR (500 MHz, CDCl_3_) δ 8.44 (d, *J* = 8.0,1H), 7.99 (br, 1H), 7.72 (t, *J* = 8.0, 1H), 7.66 (br, 1H), 7.59 (d, *J* = 8.0, 1H), 7.47–7.51 (m, 2H), 7.40 (br, 1H), 7.27–7.34 (m, 3H), 7.18 (br, 1H), 5.37 (br, 1H), 4.45 (br, 2H), 4.09 (br, 2H), 2.28 (br, 1H), 2.12 (br, 1H), 1.88 (br, 1H), 0.96 (d, *J* = 7.5, 3H). ^13^C NMR (125 MHz, CDCl_3_) δ 170.4, 146.7, 141.6, 138.6, 138.4, 130.9, 127.7, 127.3, 127.2, 126.9, 126.8, 123.1, 122.4, 110.6, 100.0, 60.7, 44.2, 25.9, 11.7. HRMS calcd for C_27_H_23_N_5_O_2_S_2_ 513.1293; found 513.1303.

#### 1-(10*H*-phenothiazin-10-yl)-2-((5(prop-2-yn-1-yl)-5*H*-[1,2,4]triazino[5,6-*b*]indol-3-yl)thiol)butan-1-one (31)

Compound **31** was synthesized similarly to **28** as amorphous powder. ^1^H NMR (500 MHz, CDCl_3_) δ 8.48 (d, *J* = 7.5 1H), 7.59 (br, 1H), 7.75–7.78 (m, 1H), 7.67–7.70 (m, 2H), 7.51–7.55 (m, 2H), 7.40 (br, 1H), 7.31–7.34 (m, 3H), 7.17 (br, 1H), 5.43 (t, *J* = 6.5, 1H), 5.08–5.13 (m, 2H), 2.40 (s, 1H), 1.92 (br, 1H), 1.89 (br, 1H), 0.92–1.01 (m, 3H). HRMS calcd for C_28_H_21_N_5_O_2_S_2_ 507.1188; found 507.1188.

#### 2-((5(but-3-yn-1-yl)-5*H*-[1,2,4]triazino[5,6-*b*]indol-3-yl)thiol)-1-(10*H*-phenothiazin-10-yl)butan-1-one (32)

Compound **32** was synthesized similarly to **28** as amorphous powder. ^1^H NMR (500 MHz, CDCl_3_) δ 8.46 (d, *J* = 7.5, 1H), 7.97 (br, 1H), 7.68–7.74 (m, 2H), 7.48–7.59 (m, 3H), 7.40 (br, 1H), 7.31–7.34 (m, 2H), 7.18 (br, 1H), 5.41 (t, *J* = 7.0, 1H), 4.45–4.52 (m, 2H), 2.77–2.80 (m, 2H), 2.14 (br, 1H), 1.89 (br, 1H), 0.98 (br, 3H). ^13^C NMR (125 MHz, CD_3_Cl) δ 169.4, 165.9, 145.8, 140.9, 140.6, 137.9, 137.8, 130.9, 128.2, 127.8, 127.5, 127.2, 127.0, 123.0, 121.5, 117.3, 111.6, 80.6, 73.3, 25.7, 17.5, 11.3. HRMS calcd for C_28_H_21_N_5_O_2_S_2_ 507.1188; found 507.1188.

#### 2-((5-(2-oxo-2-phenylethyl)-5*H*-[1,2,4]triazino[5,6-*b*]indol-3-yl)thiol)-1-(10*H*-phenothiazin-10-yl)butan-1-one (33)

Compound **33** was synthesized similarly to **28** as amorphous powder. ^1^H NMR (500 MHz, CDCl_3_) δ 8.49 (d, *J* = 7.5, 1H), 8.05 (d, *J* = 7.5, 1H), 7.90 (br, 1H), 7.65–7.72 (m, 3H), 7.55–7.58 (m, 2H), 7.49–7.52 (m, 2H), 7.40–7.46 (m, 2H), 7.31–7.39 (m, 1H), 7.21–7.24 (m, 4H), 5.69–5.77 (m, 2H), 5.39 (t, *J* = 6.5, 1H), 2.05 (br, 1H), 1.87 (d, *J* = 5.5, 1H), 0.90–0.92 (m, 3H). HRMS calcd for C_33_H_25_N_5_O_2_S_2_ 587.1450; found 507.1461.

#### 2-((5-((1-phenethyl-1H-1,2,3-triazol-4-yl)methyl)-5*H*-[1,2,4]triazino[5,6-*b*]indol-3-yl)thiol)-1-(10*H*-henothiazin-10-yl)butan-1-one (34)

2-bromoethylbenzene (3.083 g, 0.3 mmol) was dissolved in 17 ml anhydrous DMF. NaN_3_ (2.1664 g, 33.329 mmol) and 56 mg KI were added to the above solution. The reaction mixture was heated to 90°C for 18 h. TLC indicated that there was no starting material remained. The reaction was stopped and 200 ml DCM was added to the mixture. The organic phase was washed by 50 ml water and dried by anhydrous Na_2_SO_4_. The organic phase was concentrated and evaporated under vacuum. The crude azide was obtained and used for the next step directly.

Compound **32** (0.1268 g, 0.25 mmol) and azide (33.4 mg, 0.227 mmol) were dissolved in 3.6 ml *t*-BuOH/H_2_O/THF (v/v-1∶1∶1). Sodium ascorbate (98.9 mg, 0.4994 mmol) and CuSO_4_ (11.3 mg, 0.0454 mmol) in 0.5 ml water were added to the above reaction mixture. The reaction mixture was heated to 55°C. After stirring for 24 h, TLC indicated that there was no starting material remained. The reaction was stopped and cooled to room temperature. 6 ml water was added to the mixture. The solid was collected and washed with a few water. Then, the solid was dissolved in 8 ml acetone and the solution was filtered. The filtrate was evaporated and the residue was dissolved in 3 ml ethyl acetate. The solution was heated and 5 ml hexane was added to the solution. After overnight, gray solid was formed and collected. The amorphous solid **34** was washed by 4 ml hexane and dried. ^1^H NMR (500 MHz, CDCl_3_) δ 8.44 (d, *J* = 8.0, 1H), 8.03 (br, 1H), 7.66–7.77 (m, 3H), 7.48–7.51 (m, 2H), 7.41 (br, 1H), 7.32–7.33 (m, 2H), 7.18 (br, 1H), 7.06–7.13 (m, 4H), 6.93–6.95 (m, 2H), 5.46–5.56 (m, 3H), 4.53 (t, *J* = 7.5, 2H), 3.14 (t, *J* = 7.5?, 2H), 2.07 (br, 1H), 1.88 (br, 1H), 0.98 (br, 3H). HRMS calcd for C_36_H_30_N_8_OS_2_ 654.1984; found 654.1991.

#### 2-((5-((1-phenethyl-1H-1,2,3-triazol-4-yl)ethyl)-5*H*-[1,2,4]triazino[5,6-*b*]indol-3-yl)thiol)-1-(10*H*-phenothiazin-10-yl)butan-1-one (35)

Compound **35** was synthesized similarly to **34** as amorphous powder. ^1^H NMR (500 MHz, CDCl_3_) δ 8.43 (d, *J* = 7.5, 1H), 8.00 (br, 1H), 7.64–7.67 (m, 2H), 7.42–7.50 (m, 4H), 7.23–7.33 (m, 6H), 7.18 (br, 1H), 7.00–7.02 (m, 2H), 6.92 (s, 1H), 5.44 (t, *J* = 6.5, 1H), 4.62 (t, *J* = 7.0, 2H), 4.48 (t, *J* = 7.5, 2H), 3.24 (t, *J* = 7.0, 2H), 3.06 (t, *J* = 7.5, 2H), 2.04–2.08 (m, 1H), 1.87–1.89 (m, 1H), 0.92–1.01 (m, 3H). HRMS calcd for C_37_H_32_N_8_OS_2_ 668.2140; found 668.2135.

#### Ethyl 4-(4-(2-(3-((1-oxo-1-(10*H*-phenothiazin-10-yl)butan-2-yl)thio)-5*H*-[1,2,4]triazino[5,6-*b*]indol-5-yl)ethyl)-1*H*-1,2,3-triazol-1-yl)butanoate (36)

Compound **36** was synthesized similarly to **34** as amorphous powder. ^1^H NMR (500 MHz, CDCl_3_) δ 8.41 (d, *J* = 8.0, 1H), 8.01 (br, 1H), 7.62–7.68 (m, 2H), 7.50 (d, *J* = 7.5, 1H), 7.40–7.44 (m, 3H), 7.26–7.33 (m, 3H), 7.17 (br, 2H), 5.44 (t, *J* = 6.5, 1H), 4.66 (t, *J* = 7.0, 2H), 4.32 (t, *J* = 7.0, 2H), 4.15 (q, *J* = 7.0, 2H), 3.29 (t, *J* = 7.0, 2H), 2.20 (t, *J* = 7.5, 2H), 2.07–2.11 (m, 2H), 1.87 (br, 1H), 1.77 (br, 1H), 1.28 (t, *J* = 7.0, 3H), 0.94–1.00 (m, 3H). HRMS calcd for C_35_H_34_N_8_O_3_S_2_ 678.2195; found 678.2197.

#### 1-(acridin-10(9*H*)-yl)-2-((5-(2-hydroxyethyl)-5*H*-[1,2,4]triazino[5,6-*b*]indol-3-yl)thio)butan-1-one (37)

Compound **37** was synthesized similarly to **30** as viscous oil. 1H NMR (500 MHz, CDCl_3_) δ 8.35 (d, *J* = 7.5, 1H), 7.77 (br, 2H), 7.66–7.70 (m, 1H), 7.55 (d, *J* = 8.0, 1H), 7.44 (t, *J* = 8.0, 1H), 7.24–7.29 (m, 4H), 7.16 (br, 2H), 5.47 (br, 1H), 4.33–4.41 (m, 2H), 4.05–4.08 (m, 2H), 3.86 (br, 2H), 2.13–2.19 (m, 1H), 1.98–1.99 (m, 1H), 1.04 (br, 3H). HRMS calcd for C_28_H_25_N_5_O_2_S 495.1729; found 495.1731.
